# Supplementary material for: Promoting Health via mHealth Applications Using a French Version of the Mobile App Rating Scale: Adaptation and Validation Study
Source: JMIR Mhealth Uhealth. 2021 Aug 31;9(8):e30480. doi: 10.2196/30480 (PMC8441605; doi:10.2196/30480)
Supplement: Multimedia Appendix 1 [file mhealth_v9i8e30480_app1.pdf]

Multimedia Appendix 1. French version of the Mobile Application Rating Scale (MARS-F).

Échelle d'Évaluation des Applications Mobiles (MARS-F)

Classification des applications mobiles

La section Classification est utilisée pour recueillir des informations descriptives et techniques concernant l'application mobile. Veuillez consulter la description de l'application dans iTunes / Google Play pour accéder à ces informations.

Nom de l'application mobile : ____

Note de cette version : _________________ **Note de toutes les versions :** ________________

Développeur : ____

Nombre d'utilisateurs ayant évalué cette version : _____________________________________

Nombre d'utilisateurs ayant évalué toutes les versions : ________________________________

Version : ____________________________ **Dernière mise à jour :** _____________________

Coût de la version de base : _____________ **Coût de la version actualisée :** ______________

Plateforme : ☐ **iPhone** ☐ **iPad** ☐ **Android**

Brève description de l'application mobile : ____

____

Objectifs-cibles de l'application (plusieurs choix possibles) :

☐ Bonheur / Bien-être
☐ Pleine conscience / Méditation / Relaxation
☐ Emotions négatives
☐ Dépression
☐ Anxiété / stress
☐ Colère
☐ Changement de comportement
☐ Consommation d'alcool / de substances
☐ Défi personnel
☐ Divertissement
☐ Relations sociales
☐ Santé physique
☐ Autre : __________________________

Contexte théorique / Stratégies utilisées par l'application mobile (plusieurs choix possibles) :

☐ Évaluation
☐ Retour d'expérience
☐ Information / Éducation
☐ Surveillance / Suivi
☐ Objectif à atteindre
☐ Conseils / Astuces / Stratégies / Entraînement des compétences
☐ Thérapie cognitivo-comportementale (TCC) - Comportement (événements positifs)
☐ Thérapie cognitivo-comportementale (TCC) - Cognitif (stimulation par la pensée)
☐ Thérapie d'engagement et d'acceptation
☐ Pleine conscience / Méditation
☐ Relaxation
☐ Reconnaissance
☐ Basée sur les forces
☐ Autre: __________________________

Affiliations :

- ☐ Inconnue
- ☐ Commerciale
- ☐ Gouvernement
- ☐ Organisation non gouvernementale (ONG)
- ☐ Université

Groupe d'âge du public ciblé (plusieurs choix possibles) :

- ☐ Enfants (moins de 12 ans
- ☐ Adolescents (13-17 ans)
- ☐ Jeunes adultes (18-25 ans)
- ☐ Adultes

Aspects techniques de l'application (plusieurs choix possibles) :

- ☐ Autorise le partage (Facebook, Twitter, etc.)
- ☐ Dispose d'une communauté liée à l'application
- ☐ Possibilité de protéger par mot de passe
- ☐ Requiert un identifiant de connexion
- ☐ Envoie des rappels
- ☐ Besoin d'un accès internet pour fonctionner

Evaluation de la qualité de l'application mobile

L'échelle évalue la qualité de l'application sur 4 sections (A à D). Toutes les réponses sont évaluées sur 5 modalités de "1. Inadéquat" à "5. Excellent". Entourez la réponse qui représente le plus précisément possible la qualité de l'application évaluée. Veuillez utiliser la nomenclature fournie pour chaque catégorie de réponse.

SECTION A - Engagement

Amusant, intéressant, personnalisable, interactif (par exemple, envoie des alertes, des messages, des rappels, des retours d'expérience, permet le partage), sur un public ciblé

1. **Divertissement : l'application est-elle amusante / divertissante à utiliser ? Utilise-t-elle des stratégies pour augmenter l'engagement grâce au divertissement (par exemple, grâce à la ludification) ?**

    1. Terne, pas du tout amusante ou divertissante
    2. Plutôt ennuyeuse
    3. OK, assez amusante pour divertir l'utilisateur pendant un bref instant (<5 minutes)
    4. Modérément amusante et divertissante, divertirait l'utilisateur pendant un certain temps (5 à 10 minutes au total)
    5. Très divertissante et amusante, stimulerait une utilisation répétée

2. **Intérêt : l'application est-elle intéressante à utiliser ? Utilise-t-elle des stratégies pour accroître l'engagement en présentant son contenu de manière intéressante ?**

    1. Pas du tout intéressante
    2. Plutôt inintéressante
    3. OK, ni intéressante ni inintéressante ; engagerait l'utilisateur pendant une courte durée (<5 minutes)
    4. Modérément intéressante ; engagerait l'utilisateur pendant un certain temps (5 à 10 minutes au total)
    5. Très intéressante, engagerait l'utilisateur de manière répétée

3. **Personnalisation : l'application fournit-elle ou conserve-t-elle tous les paramètres / préférences nécessaires pour ses fonctionnalités (par exemple : sons, contenu, notifications, etc.)?**

    1. N'autorise aucune personnalisation ou nécessite la saisie d'un réglage à chaque fois
    2. Permet une personnalisation insuffisante limitant les fonctionnalités
    3. Permet une personnalisation de base pour un fonctionnement adéquat
    4. Permet de nombreuses options de personnalisation
    5. Permet une adaptation complète aux caractéristiques / préférences de l'utilisateur, conserve tous les paramètres

4. **Interactivité : l’application permet-elle à l'utilisateur d'intervenir, d’apporter un retour d'information, de contenir des invitations (rappels, options de partage, notifications, etc.)? Remarque : ces fonctionnalités doivent être personnalisables et non imposées pour être parfaites.**

    1. Aucune fonctionnalité interactive et / ou aucune réponse à l'interaction de l'utilisateur
    2. Interactivité insuffisante, ou retour d’expérience, ou options de saisie de l’utilisateur, limitant les fonctionnalités
    3. Fonctionnalités interactives de base pour un fonctionnement adéquat
    4. Offre une variété de fonctionnalités interactives / retour d’expérience / options de saisie de l’utilisateur
    5. Très haut niveau de réactivité grâce aux fonctionnalités interactives / retour d’expérience / options de saisie de l’utilisateur

5. **Groupe ciblé : le contenu de l'application (informations visuelles, langage, conception) est-il adapté à votre public cible ?**

    1. Totalement inapproprié / peu clair / déroutant
    2. Généralement inapproprié / peu clair / déroutant
    3. Acceptable mais non ciblé. Peut-être inapproprié / peu clair / déroutant
    4. Bien ciblé, avec des problèmes négligeables
    5. Parfaitement ciblé, aucun problème détecté

Score moyen de la section A – Engagement = ____

SECTION B - Fonctionnalité

Fonctionnement de l'application, facile à apprendre, navigation, logique de flux, et conception gestuelle de l'application

6. **Performance : avec quelle précision / rapidité les fonctionnalités et les composants de l'application (boutons / menus) fonctionnent-ils ?**

    1. L'application ne fonctionne pas ; pas de réponse / réponse insuffisante / réponse imprécise (par exemple, plantages / bugs / fonctionnalités cassées, etc.)
    2. Certaines fonctionnalités fonctionnent, mais sont lentes ou présentent des problèmes techniques majeurs
    3. L'application fonctionne dans l'ensemble. Certains problèmes techniques doivent être corrigés / parfois lents
    4. Principalement fonctionnelle avec des problèmes mineurs / négligeables
    5. Réponse parfaite / rapide ; aucun bug technique trouvé / contient un indicateur « temps de chargement restant »

7. **Facilité d'utilisation : dans quelle mesure est-il facile d'apprendre à utiliser l'application ; dans quelle mesure les étiquettes/icônes des menus et les instructions sont-elles claires?**

    1. Pas d'instruction / instructions limitées ; les étiquettes / icônes de menu sont déroutantes ; compliquées
    2. Utilisable après beaucoup de temps / d'efforts
    3. Utilisable après un certain temps / effort
    4. Facile d’apprendre à utiliser l'application (ou les instructions sont claires)
    5. Capable d'utiliser l'application immédiatement ; intuitif ; facile

8. **Navigation : le déplacement entre les écrans est-il logique / précis / approprié / ininterrompu ? Tous les liens nécessaires de navigation entre les écrans sont-ils présents ?**

    1. Différentes sections de l'application semblent sans suites logiques et aléatoires / déroutantes / la navigation est difficile
    2. Utilisable après beaucoup de temps / d'efforts
    3. Utilisable après un certain temps / effort
    4. Facile à utiliser ou manque un lien négligeable
    5. Navigation parfaitement logique, simple, claire et intuitive, fluidité ou offre des raccourcis

9. **Conception gestuelle : les interactions (tapotements / balayages / pincements / défilement) sont-elles cohérentes et intuitives à travers tous les composants / écrans ?**

    1. Complètement incohérentes / déroutantes
    2. Souvent incohérentes / déroutantes
    3. OK avec quelques incohérences / éléments déroutants
    4. Principalement cohérentes / intuitives avec des problèmes négligeables
    5. Parfaitement cohérentes et intuitives

Score moyen de la section B – Fonctionnalité = ____

SECTION C - Esthétique

Conception graphique, attrait visuel global, jeu de couleurs et cohérence stylistique.

10. Mise en page : la disposition et la taille des boutons / icônes / menus / contenu sur l'écran, sont-elles appropriées ou peuvent-elles être zoomées si nécessaire ?

1. Très mauvaise conception, encombrée, certaines options impossibles à sélectionner / localiser / voir / lire, l'affichage de l'appareil non optimisé
2. Mauvaise conception, aléatoire, peu claire, certaines options difficiles à sélectionner / localiser / voir / lire
3. Satisfaisant, quelques problèmes de sélection / localisation / visualisation / lecture des éléments ou problèmes mineurs de taille d'écran
4. Plutôt clair, capable de sélectionner / localiser / voir / lire des éléments
5. Professionnel, simple, clair, ordonné, organisé de manière logique, affichage optimisé sur le dispositif. Chaque élément de conception a un objectif.

11. Graphismes : quelle est la qualité / résolution des graphismes utilisés pour les boutons / icônes / menus / contenu ?

1. Graphismes amateurs, conception visuelle très médiocre - disproportionnée, complètement incohérente sur le plan stylistique
2. Graphismes de faible qualité / faible résolution ; conception visuelle de mauvaise qualité - disproportionnée, incohérente sur le plan stylistique
3. Graphismes et conception visuelle de qualité moyenne (style généralement cohérent)
4. Graphismes de haute qualité / résolution et conception visuelle - principalement proportionnés, stylistiquement cohérents
5. Graphismes et conception visuelle de très haute qualité / résolution - proportionnés, cohérents d'un point de vue stylistique

12. Attrait visuel : quelle est la qualité de l'application ?

1. Aucun attrait visuel, désagréable à regarder, mal conçue, couleurs contrastées / dépareillées
2. Peu d'attrait visuel - mal conçu, mauvaise utilisation de la couleur, visuellement ennuyeux
3. Un certain attrait visuel - moyen, ni agréable ni désagréable
4. Haut niveau d'attrait visuel - graphiques homogènes - conception cohérente et professionnelle
5. Comme ci-dessus + très attrayant, mémorable, remarquable ; l'utilisation de la couleur améliore les fonctionnalités / menus de l'application

Score moyen de la section C - Esthétique = ____

SECTION D - Information

Contient des informations de haute qualité (par exemple du texte, des retours d'expérience, des mesures, des références) provenant d'une source crédible. Sélectionnez N/A si la question n'est pas pertinente.

13. Précision de la description de l'application (dans la boutique d'applications) : l'application contient-elle ce qui est décrit ?

1. Trompeuse. L'application ne contient pas les composants / fonctionnalités décrites ou n'a pas de description
2. Imprécise. L'application contient très peu de composants / fonctionnalités décrites
3. OK. L'application contient certains des composants / fonctionnalités décrites
4. Exacte. L'application contient la plupart des composants / fonctionnalités décrites
5. Description très précise des composants / fonctionnalités de l'application

14. Objectifs : l'application a-t-elle des objectifs spécifiques, mesurables et réalisables (spécifiés dans la description de la boutique d'applications ou dans l'application elle-même) ?

N/A La description ne répertorie pas les objectifs, ou les objectifs de l'application ne sont pas pertinents pour l'objectif de la recherche (par exemple, utiliser un jeu à des fins éducatives).
1. L'application n'a aucune chance d'atteindre les objectifs fixés
2. La description répertorie certains objectifs, mais l'application a très peu de chances de les atteindre
3. Ok. L'application a des objectifs clairs, qui peuvent être réalisables.
4. L'application a des objectifs clairement définis, mesurables et réalisables
5. L'application a des objectifs spécifiques et mesurables, qui ont de fortes chances d'être atteints

15. Qualité des informations : le contenu de l'application est-il correct, bien rédigé et pertinent par rapport à l'objectif / au sujet de l'application ?

N/A Il n'y a aucune information dans l'application
1. Non pertinent / inapproprié / incohérent / incorrect
2. Pauvre. Très peu pertinent / approprié / cohérent / peut être incorrect
3. Modérément pertinent / approprié / cohérent / et semble correct
4. Pertinent / approprié / cohérent / correct
5. Très pertinent, approprié, cohérent et correct

16. Quantité d'informations : le contenu est-il en phase avec les objectifs annoncés de l'application - compréhensif mais concis ?

N/A Il n'y a aucune information dans l'application
1. Minimal ou accablant
2. Insuffisant ou éventuellement accablant
3. OK mais pas complet ou concis
4. Offre un large éventail d'informations, présente quelques lacunes ou des détails inutiles ; ou n'a aucun lien vers plus d'informations et de ressources
5. Complet et concis ; contient des liens vers plus d'informations et de ressources

17. **Informations visuelles : l'explication visuelle des concepts - à travers des tableaux / graphiques / images / vidéos, etc. - est-elle claire, logique, correcte ?**

N/A Il n'y a pas d'informations visuelles dans l'application (par exemple, elle ne contient que de l'audio ou du texte)
1. Pas du tout clair / déroutant / faux ou nécessaire mais manquant
2. Généralement peu clair / déroutant / faux
3. OK mais souvent peu clair / déroutant / faux
4. Généralement clair / logique / correct avec des problèmes négligeables
5. Parfaitement clair / logique / correct

18. **Crédibilité : l'application provient-elle d'une source légitime (spécifiée dans la description de la boutique des applications ou dans l'application elle-même) ?**
1. Source identifiée mais la légitimité / la fiabilité de la source est discutable (par exemple, entreprise commerciale avec un intérêt direct)
2. Semble provenir d'une source légitime, mais ne peut pas être vérifiée (par exemple, n'a pas de page Web)
3. Développée par une ONG / institution (hôpital, etc.) / entreprise commerciale spécialisée, organisme de financement
4. Développée par un gouvernement, une université ou comme ci-dessus mais à plus grande échelle
5. Développée à l'aide d'un financement gouvernemental ou de recherche compétitif au niveau national

19. **Preuves scientifiques : l'application a-t-elle été testée/évaluée ; doit être vérifiée par des preuves (dans la littérature scientifique publiée) ?**

N/A L'application n'a pas été testée/évaluée
1. Les preuves suggèrent que l'application ne fonctionne pas.
2. L'application a été testée (par exemple, acceptabilité, utilisabilité, taux de satisfaction) et a des résultats partiellement positifs dans des études qui ne sont pas des essais contrôlés randomisés (ECR), ou il existe peu ou pas de preuves contradictoires.
3. L'application a été testée (par exemple, acceptabilité, utilisabilité, taux de satisfaction) et a des résultats positifs dans des études qui ne sont pas des essais contrôlés randomisés (ECR), et il n'y a pas de preuves contradictoires.
4. L'application a été testée et les résultats ont été évalués dans 1 à 2 essais contrôlés randomisés (ECR) indiquant des résultats positifs.
5. L'application a été testée et les résultats testés dans plus de 3 essais contrôlés randomisés (ECR) de haute qualité indiquant des résultats positifs.

Score moyen de la section D - Information = ____

Qualité subjective de l'application

SECTION E

---

20. Recommanderiez-vous cette application aux personnes qui pourraient en bénéficier ?

1. Pas du tout, je ne recommanderais cette application à personne
2. Il y a très peu de personnes à qui je recommanderais cette application
3. Peut-être qu'il y a plusieurs personnes à qui je la recommanderais
4. Je recommanderais cette application à de nombreuses personnes
5. Je recommanderais certainement cette application à tout le monde

21. Combien de fois pensez-vous que vous utiliseriez cette application au cours des 12 prochains mois si elle était pertinente pour vous ?

1. Aucune
2. 1-2
3. 3-10
4. 10-50
5. >50

22. Payeriez-vous pour cette application ?

1. Non
3. Peut-être
5. Oui

23. Quelle est votre note globale en étoiles sur l'application ?

1. ☆ L'une des pires applications que j'ai utilisées
2. ☆☆
3. ☆☆☆ Moyenne
4. ☆☆☆☆
5. ☆☆☆☆☆ L'une des meilleures applications que j'ai utilisées

Score moyen de la section E – Qualité = ____

Synthèse de l'évaluation :

Sections

A : Engagement Score moyen = ____
B : Fonctionnalité Score moyen = ____
C : Esthétique Score moyen = ____
D : Information Score moyen = ____
E : Qualité Score moyen = ____

Qualité de l'application : Score moyen = ____
Qualité subjective de l'application : Score moyen = ____

SECTION F - Spécificités de l'application

Ces éléments ajoutés peuvent être ajustés et utilisés pour évaluer l'impact perçu de l'application sur les connaissances, les attitudes, les intentions de changement de l'utilisateur ainsi que la probabilité du changement réel du comportement cible en matière de santé.

Sensibilisation : cette application est susceptible de sensibiliser davantage à l'importance de lutter contre [insérer le comportement cible en matière de santé].

Pas du tout d'accord				Tout à fait d'accord
1	2	3	4	5
○	○	○	○	○

Connaissances : cette application est susceptible d'améliorer les connaissances / la compréhension de [insérer le comportement de santé cible].

Pas du tout d'accord				Tout à fait d'accord
1	2	3	4	5
○	○	○	○	○

Attitudes : cette application est susceptible de changer les attitudes vis-à-vis de l'amélioration de [insérer le comportement cible en matière de santé]

Pas du tout d'accord				Tout à fait d'accord
1	2	3	4	5
○	○	○	○	○

Intention de changer : cette application est susceptible d'augmenter les intentions / la motivation à l'égard de [insérer le comportement de santé cible]

Pas du tout d'accord				Tout à fait d'accord
1	2	3	4	5
○	○	○	○	○

Recherche d'aide : l'utilisation de cette application est susceptible d'encourager la recherche d'une aide supplémentaire pour [insérer le comportement de santé cible] (si cela est nécessaire).

Pas du tout d'accord				Tout à fait d'accord
1	2	3	4	5
○	○	○	○	○

Changement de comportement : l'utilisation de cette application est susceptible d'augmenter / diminuer [insérer le comportement de santé cible]

Pas du tout d'accord				Tout à fait d'accord
1	2	3	4	5
○	○	○	○	○
