# Supplementary material for: Promoting Health via mHealth Applications Using a French Version of the Mobile App Rating Scale: Adaptation and Validation Study
Source: JMIR Mhealth Uhealth. 2021 Aug 31;9(8):e30480. doi: 10.2196/30480 (PMC8441605; doi:10.2196/30480)
Supplement: Multimedia Appendix 3 [file mhealth_v9i8e30480_app3.pdf]

Multimedia Appendix 3. Comparison of internal consistencies and the Mokken Scale Analysis between Mobile App Rating Scale in English and other available translations of the scale.

			MARS English	MARS German	MARS Italian	MARS Spanish	MARS Arabic	MARS French
	Intraclass Correlation (95%CI)		0.79 (0.75-0.83)	0.83 (0.82-0.85)	0.96 (0.93-0.98)	0.96 (0.92-0.98)	0.84 (0.82-0.85)	0.89 (0.80-0.93)
**Internal consistency**	Engagement	Cronbach's alpha (95%CI)	0.89		Rater1=0.85 (0.76-0.91) Rater2=0.84 (0.75-0.90)	Rater1=0.88 Rater2=0.87	0.96	
		Omega coefficient (95%CI)		0.84 (0.77-0.88)				0.79 (0.72-0.83)
	Functionality	Cronbach's alpha (95%CI)	0.80		Rater1=0.77 (0.63-0.87) Rater2=0.87 (0.79-0.92)	Rater1=0.87 Rater2=0.86	0.71	
		Omega coefficient (95%CI)		0.90 (0.85-0.94)				0.79 (0.73-0.85)
	Esthetics	Cronbach's alpha (95%CI)	0.86		Rater1=0.92 (0.86-0.95) Rater2=0.88 (0.81-0.93)	Rater1=0.86 Rater2=0.77	0.94	
		Omega coefficient (95%CI)		0.91 (0.92-0.96)				0.78 (0.71-0.82)
	Information quality	Cronbach's alpha (95%CI)	0.81		Rater1=0.73 (0.57-0.84) Rater2=0.71 (0.54-0.83)	Rater1=0.84 Rater2=0.78	0.81	
		Omega coefficient (95%CI)		0.74 (0.14-0.99)				0.61 (0.53-0.65)
	Total score	Cronbach's alpha (95%CI)	0.85 (0.80-0.89)		Rater1=0.90 (0.85-0.94) Rater2=0.91 (0.87-0.94)	Rater1=0.94 Rater2=0.94		
		Omega coefficient (95%CI)		0.81 (0.74-0.86)				0.86 (0.85-0.90)
	Subjective quality	Cronbach's alpha (95%CI)	0.93		Rater1=0.95 (0.92-0.97) Rater2=0.93 (0.89-0.96)	Rater1=0.86 Rater2=0.88	0.97	
Mokken Scale Analysis				H=0.48				H=0.35;
				SE 0.06				SE 0.03
				MS=0.74				MS=0.88
				lambda 2=0.72				lambda 2=0.89
				LCRC=0.74				LCRC=0.90
